# Hydrolytically Stable Cationic Bis-MPA Dendrimers as Efficient Transfectants for Glioblastoma Cells and Primary Astrocytes

**DOI:** 10.1021/acs.biomac.5c01202

**Published:** 2025-12-03

**Authors:** Angel Buendía, Natalia Sanz Del Olmo, Irene Rodríguez-Clemente, Jacob Wohlert, Krzysztof Sztandera, Jorge San Jacinto García, Faridah Namata, Michael Malkoch, Valentín Ceña

**Affiliations:** † Unidad Asociada Neurodeath, Institute of Molecular Nanoscience, INAMOL, School of Medicine, 16733 Universidad de Castilla-La Mancha, Albacete 02006, Spain; ‡ CIBERNED, CIBER, Instituto de Salud Carlos III, Madrid 28029, Spain; § Department of Fiber and Polymer Technology, 7655 KTH Royal Institute of Technology, Teknikringen 56-68, 100 44 Stockholm, Sweden; ∥ Wallenberg Wood Science Centre (WWSC), KTH Royal Institute of Technology, 100 44 Stockholm, Sweden

## Abstract

We report the biological evaluation of bis-MPA dendrimers terminated with either cysteamine (CYS) or 2-(dimethylamino)­ethanethiol (DA) groups for siRNA transfection. The results show that aggregation phenomena are critical to the biological performance of these constructs. Confocal and 2D microscopy demonstrated that only the G3-CYS dendrimer transported siRNA into cells. Accordingly, G3-CYS-mediated siRNA transfection reduced intracellular levels of the target proteins—p42-MAPK, Rheb, and MGMT—to 15–25% of control levels in a human glioblastoma cell line and mouse astrocytes. G3-CYS transfection efficiency was similar to that of commercial transfectants. However, its self-degradable bis-MPA backbone and tunable peripheral groups render it markedly superior, making it a promising transfection agent and emphasize the critical balance between structural design, biological efficacy, and safety. Despite its efficacy, G3-CYS displayed a narrow therapeutic window with pronounced cytotoxicity above 1 μM. In vivo studies further confirmed dose-dependent systemic toxicity, likely associated with enhanced blood coagulation.

## Graphical abstract

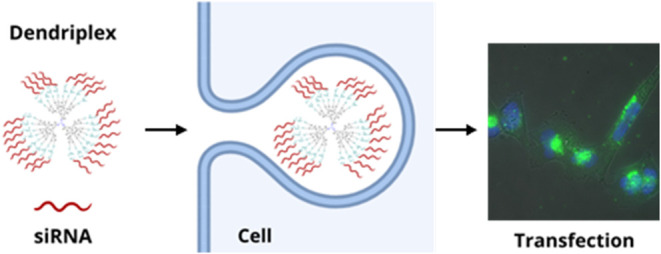

## Introduction

Glioblastomas (GBMs) are the most common brain tumors in adults^1^ characterized by their highly infiltrative and diffuse nature.^2^ Current primary GBM treatment includes a combination of surgical resection, radiotherapy, and Temozolomide (TMZ) chemotherapy.^3^ Despite these approaches, the prognosis remains poor,^4^ with an overall survival rate of approximately 20% two years postdiagnosis.^5^ This underscores the urgent need for novel therapeutic strategies for this deadly disease.

RNA interference (RNAi) is a mechanism that regulates cellular metabolism, replication and malignant transformation in most eukaryotic cells.^6^ Small interfering RNA (siRNA), a synthetic double-stranded RNA of 20 to 24 base pairs, effectively suppresses target protein expression, leading to interference with various mechanisms involved in the pathogenesis of several diseases. It is highly specific for its target and can markedly reduce the target protein levels in short time.^7^ However, naked siRNA is highly labile and prone to rapid degradation by RNases, necessitating the use of nonviral cationic carriers for protection and intracellular delivery.^8^ Several siRNA-based treatments have already reached the clinical setting.^9^

Nonviral vectors, including lipids, polymers, inorganic particles, and dendrimers, possess advantages such as stability, tunable surface properties, and reduced immunogenicity, making them promising biomedical tools. Among these, cationic dendrimers stand out as potential siRNA carriers.^10^ These monodisperse macromolecules exhibit uniform size and shape, internal cavities for molecular encapsulation, and a high density of tunable surface functional groups.^11^ Such features enable their use in diverse biomedical applications.^10^

A major challenge with nanoparticles, including dendrimers, is their potential intracellular accumulation, leading to toxicity—an issue particularly relevant for carbon-based nanoparticles.^12^ This has driven research toward biodegradable nanoparticles. In this context, 2,2-bis­(hydroxymethyl)­propionic acid (bis-MPA) dendrimers have emerged as promising candidates, demonstrating nontoxicity and biodegradability.^13^ Additionally, β-alanine-functionalized bis-MPA dendrimers exhibit antimicrobial properties due to their cationic nature.^14^ In 2018, Malkoch’s group reported bis-MPA polyester-based dendrimers with trimethylolpropane (TMP) cores, functionalized with β-alanine, showing promising siRNA transfection results in glioma C6 cell lines.^14^ The third and fourth generation dendrimers, with 12 and 24 peripheral cationic groups, respectively, showed promising in vitro inhibition of 20% of target protein p42-MAPK expression in glioma (C6) cell lines.^14^ However, despite these encouraging transfection results, based on β-alanine functionalized bis-MPA dendrimers, their degradation rate at physiological pHs was very high, compromising long-term effectiveness.^13^

While biodegradability is a highly desirable feature in biomedical applications, excessive degradation can compromise long-term effectiveness. Therefore, it is crucial to develop systems with a more prolonged degradation profile. To increase hydrolytic stability while keeping the peripheral cationic amines, the outer layer of bis-MPA dendrimers was modified using thiol–ene click chemistry (TEC) to introduce thioether bonds, resulting in cysteamine HCl (Cys) and 2-(dimethylamino)­ethanethiol HCl (DA) functionalized dendrimers. These dendrimers exhibited high stability at physiological pH for up to one-month, excellent antibacterial activity, and good biocompatibility at the concentrations effective against both Gram-positive and Gram-negative bacteria.^15^

In this study, we explore the therapeutic potential of hydrolytically stable, cationic bis-MPA dendrimers as nonviral vectors for siRNA delivery against glioblastoma. We assess their siRNA complexation ability, protection from RNases, and the toxicity of the cationic vector in a commercial human GBM cell line (T98G). To assess their versatility and translational relevance, in vitro transfection efficiency was further evaluated in primary mouse astrocytes. As a proof of concept for real-time tracking and biodistribution, the third-generation dendrimer was fluorescently labeled with Cyanine 7.5 and examined in vivo. We also studied the dendrimer effect on blood coagulation. Finally, molecular dynamics simulations and particle size analyses were conducted to gain deeper insights into their structural behavior in aqueous environments.

## Experimental Section

### Materials

All reagents and solvents were purchased from Sigma-Aldrich and used as received unless otherwise noted. The dye Cyanine7.5 NHS ester (Cy7.5) was purchased from Lumiprobe GmbH, (Hannover, Germany).

### Synthetic Protocols

Dendrimers functionalized with cysteamine, and dimethylamine hydrochloride have been synthesized based on previously reported protocols.^15,16^ The synthesis of the third-generation dendrimer labeled with Cy7.5 can be found in the Supporting Information.

### Characterization Methods

#### Nuclear Magnetic Resonance (NMR)

Analyses were performed using a Bruker AM NMR. ^1^H NMR was recorded at 400 MHz and acquired using a spectral window of 20 ppm, a relaxation delay of 1 s, and 16 scans with automatic lock and shimming. Diffusion ordered spectroscopy (DOSY-NMR) was performed for the labeled dendrimer. Analyses of the obtained spectra were conducted using MestReNova version 14.2.0–26256 (Mestrelab Research S.L., Santiago de Compostela, Spain).

#### UV–Vis Spectrophotometer

UV–vis measurements were performed by dissolving the dendrimer in water at a concentration of 10 μM, and absorbance was measured in the range of 600–900 nm using an Infinite M200 (Tecan, Männedorf, Switzerland) plate reader.

#### Scanning Electron Microscopy (SEM)

Dendrimers were dissolved in DI water at 5 μM and deposited on top of a silica wafer to be dried overnight at room temperature. Then, samples were coated with Au–Pd thin layer (5 s coating) with a Sputter-coater-JFC1300 (JEOL, Peabody. USA). Using a high-resolution high-vacuum cold field-emission Hitachi SEM S-4800 (Hitachi, Tokio, Japan), equipped with a Secondary Electron detector (SE), Backscattered Electron detector (BSE); Scanning Transmission Electron detector (STEM); Energy Dispersive X-ray Spectrometry (EDS) detectors, samples were investigated under 1 kV acceleration voltage, a current of 2 mA and working distance of 2 mm. Pictures were taken at 2.5 k and 4.5 k magnifications.

#### Dynamic Light Scattering (DLS)

Hydrodynamic diameter for the G3-CYS dendrimer was measured using the DLS technique. The dendritic compound was dissolved in DI water as the dispersant at a concentration of 500 μM with measurements conducted at 25 °C. Each sample was allowed to equilibrate for 120 s prior to analysis. All results are averages of at least three individual samples consisting of 10 runs each. Data was processed using ZS Xplorer (2.0.1.1. Malvern Panalytical Ltd., Malvern, UK).

#### Nanoparticle Tracking Analyzer (NTA)

NTA measurements were performed using a NanoSight NS300 (Malvern Technologies, Malvern, UK) equipped with solid-state, single-mode laser diode (<20 mW, 655 nm) configured to launch a finely focused beam through a 500 μL sample chamber. TMP-G3-Cys dendrimer was dissolved in 0.2 μm-filtered PBS (Gibco, Whatman, USA) pH = 7.4 at a concentration of 10 μM and injected into the sample chamber with 1 mL syringes until the liquid reached the tip of the nozzle. The size distribution analysis was performed at 37 °C. Each sample was analyzed three times with five technical replicates using a manual screen gain and camera level adjustments to obtain the optimal visualization of the sample. The NTA software (Version 3.4 from NanoSight) was used for data acquisition and analysis with a detection threshold of 4.

### Agarose Gel Retardation

Dendrimers/scramble siRNA complexes were prepared at increasing dendrimer protonable nitrogens/siRNA phosphorus (N/P) ratios by incubation at room temperature for 30 min. Samples were then loaded onto a 1.2% agarose gel containing 0.004% (v/v) Red Safe (Intron Biotech, South Korea) in TAE buffer (40 mM Tris base, 20 mM glacial acetic acid, and 1 mM ethylenediamine tetra-acetic acid [EDTA], at pH 8.6), and the resulting gels were photographed under UV illumination.^7^ The fluorescent bands intensities were measured using ImageJ software.^17^

### siRNA Protection against RNases

Dendrimer-mediated protection against siRNA degradation by ribonuclease A (RNase) was performed as previously described.^8^ Briefly, either naked siRNA (100 nM) or the complexes, prepared as indicated above, were incubated in the presence of RNase (0.25% w/v) for 30 min at 37 °C. Samples were then cooled at 4 °C for 20 min to inactivate RNase. Heparin (1.5 IUs) was added for an additional 20 min at 4 °C to the mixture to release siRNA and the samples were processed as described above.

### Cell Culture

T98G human GBM cells were obtained from the American Type Culture Collection (ATTC, Rockville, MD, USA) and cultured according to the provider instructions. Primary astrocytes were isolated from one-day-old mouse pups as previously described.^18^ Cells were cultured in Dulbecco’s Modified Eagle’s Medium (DMEM; Thermo Fisher; Waltham, MA, USA) supplemented with 10% heat-inactivated fetal bovine serum (FBS), 2 mM l-glutamine, 100 μg/mL streptomycin, and 100 IU/mL penicillin in an incubator with humidified atmosphere containing 95% air and 5% CO2 at 37 °C. The animal experimental study was approved by the Animal Experimentation Ethics Committee at the University of Castilla-La Mancha (UCLM; protocol number PR-2014–10–12) and carried out in accordance with the guidelines from the same UCLM committee and the European Union (directive 2010/63/EU) for the use of laboratory animals.

### Cell Toxicity

Cellular toxicity of the dendrimers was studied by measuring the release of lactate dehydrogenase (LDH) to the culture medium using the CytoTox96 Non-Radioactive Cytotoxicity Assay kit (Promega, Madrid, Spain), as previously described.^8,19^ Briefly, cells were incubated for 72 h with increasing concentrations of Bis-MPA-based dendrimers poly­(amidoamine) (PAMAM) dendrimers ranging from 0.1 μM to 10 μM, or either Fugene (Thermo Fisher, Waltham, MA, USA) or Lipofectamine RNAiMAX (Thermo Fisher, Waltham, MA, USA), ranging from 100-fold lower to 10-fold higher concentrations than those recommended by the supplier (1.5 μL/well for Fugene and 1 μL/well for Lipofectamine RNAiMAX). Culture medium was collected and the cells lysed using 0.1% (w/v) Triton X-100 in NaCl (0.9%). LDH content in both culture media and cell lysates was determined using a spectrophotometer (Infinite 200, Tecan, Männedorf, Switzerland). LDH release was calculated as the LDH released/total LDH ratio, with the latter being the sum of the LDH content in the culture medium plus the cellular LDH content.

### siRNA Uptake

siRNA cellular uptake was studied as previously described.^20^ Briefly, cells were seeded on 20 mm glass coverslips and cultured in DMEM medium containing l-glutamine (2 mM), FCS (10%), penicillin, 100 IU/mL, and streptomycin, 100 μg/mL. Dendriplexes were prepared by incubating the different dendrimers (G2-DA, 1 μM; G3-DA, 1 μM; G1-CYS, 5 μM; G2-CYS, 1 μM; and G3-CYS, 1 μM) with 5′-Carboxyfluorescein (FAM)-labeled siRNA (100 nM; Merck, KGaA, Darmstadt, Germany) for 1 h at room temperature in DMEM. Next, 10% FCS, antibiotics, and l-glutamine were added to generate complete medium. The cell culture incubation medium was then replaced with medium containing the dendriplexes. Ten minutes before recording the data, Hoechst 33342 (25 μg/mL; Thermo Fisher, Waltham, MA, USA) was added to the culture medium. After 6 h of incubation, the cells were washed 3 times with Krebs–Henseleit (KH) solution (NaCl 140 mM, CaCl_2_ 2.5 mM, MgCl_2_ 1 mM, KCl 5 mM, *N*-2-hydroxyethylpiperazine-*N*′-2-ethanesulfonic acid [HEPES] 5 mM, and glucose 11 mM, at pH 7.4) and the fluorescence was recorded in a Nikon Eclipse TE2000-E fluorescence microscope (Nikon, Tokyo, Japan). Images were recorded using a 60× fluorescence, oil immersion objective, an ORCA camera (Hamamatsu, Hamamatsu City, Japan), and analyzed using NIS Elements AR software (Nikon, Tokyo, Japan). The excitation and emission wavelengths were set at 488 and 520 nm for FAM-siRNA and to 350 and 450 nm for Hoechst 33342, respectively. Intracellular fluorescence intensity quantification was performed using ImageJ software.^17^

Confocal studies were performed using a Leica Stellaris confocal microscope platform (Wetzlar, Germany) with spectral separation to discriminate between the fluorochromes and a 100x oil immersion objective. Cells were treated as indicated above, incorporating MitoTracker Green (Thermo Fisher, Waltham, MA, USA) to label mitochondria and Hoechst 33342 for nuclear staining. Z step was fixed at 0.4 μm.

### Western Blot Analysis

Western blot analysis was performed as previously described.^21^ Briefly, T98G cells or astrocytes were incubated with the different dendrimers (G2-DA, 1 μM; G3-DA, 1 μM; G1-CYS, 5 μM; G2-CYS, 1 μM; G3-CYS, 1 μM; PAMAM 1 μM) either alone or with the dendrimers/siRNA complexes formed by incubating during 1 h the corresponding dendrimer with scramble noncoding siRNA (SCR) or the specific siRNA (25, 50, and 100 nM) aimed to knock down the target protein (p42 Mitogen-Activated Protein Kinase; p42-MAPK; Ras homologue enriched in brain, Rheb; O^6^-methylguanine-DNA methyltransferase, MGMT). A similar protocol was used for the commercial transfection reagents Fugene (1.5 μL/well) or Lipofectamine RNAiMAX (1 μL/well).

The siRNAs were designed, without any base chemical modification, against the following mRNA sequences from the GenBank database (NIH, Bethesda, MD, USA):

Human p42-MAPK: Target position: 355–377. Access number: NM_138957.2

Human Rheb: Target position: 597–619. Access number: NM_005614

Human MGMT: Target position: 129–151. Access number: NM_002412

Mouse p42-MAPK: Target position: 517–537. Access number: NM_011949

Mouse Rheb: Target position: 267–289. Access number: NM_053075.3

After 72 h, the medium was removed, the cells were lysed, and 30 μg of protein were loaded onto 12% sodium dodecyl sulfate polyacrylamide gel electrophoresis (SDS-PAGE) and run at 90 V until the gel front was 0.5 cm from the bottom of the plate. The gels were then transferred to nitrocellulose membranes (Bio-Rad Laboratories, Madrid, Spain) and the immunocomplexes were visualized using an enhanced chemiluminescence system. The following primary antibodies: polyclonal against: p42-MAPK (Erk2) (1:500; Cell Signaling Technology, Leiden, The Netherlands), Rheb, (1:1000; Cell Signaling Technology, Leiden, The Netherlands), a GTPase belonging to the mammalian target of rapamycin complex 1 (MTORC1); MGMT (1:1000; Cell Signaling Technology, Leiden, The Netherlands), the enzyme that reverses the antitumoral effect of TMZ in GBM cells; and monoclonal anti-GAPDH antibody (1:2000; Cell Signaling Technology, Leiden, The Netherlands). Immunoreactive bands were quantified by densitometry using ImageJ software^17^ and the results were expressed as the ratio of the targeted protein density/GAPDH density, using the latter as a loading-control protein.

### Molecular Dynamics

An atomistic model of the G3-CYS dendrimer was build using the “Ligand Reader & Modeler” module of the CHARMM graphical user interface^22,23^ with potential parameters from the CHARMM general force field^24,25^ in combination with chloride parameters from Orabi et al. and TIP3P water.^26^ A single dendrimer was put in a cubic computational box with periodic boundary conditions and a side length of six nanometer together with 24 randomly placed chloride ions to achieve charge neutrality. This system was solvated using 6716 water molecules and equilibrated at constant room temperature and atmospheric pressure for ten nanoseconds. The equilibrated system was replicated in one direction to create a computational box of approximately 12 nm × 6 nm × 6 nm, containing two dendrimers, 48 chloride ions and 13,432 water molecules. This system was the starting point for simulations of the separated state. Next, a harmonic force with force constant 1000 kJ mol^–1^ nm^–2^ was applied on the center-of-mass distance between the dendrimers, leading to rapid aggregation. The end structure after 10 ns of simulation was used as starting point for simulations of the aggregated state. The two production runs amounted to 35 ns each. All MD simulations were performed using GROMACS 2021 ^27^ using a leapfrog algorithm with a basic time step of two fs. All bonds were kept at their equilibrium lengths using the LINCS constraint algorithm^28^ while water was kept completely rigid using SETTLE.^29^ Temperature was set to 310 K (where not stated otherwise) using stochastic velocity rescaling^30^ and pressure was maintained at 1 atmosphere using isotropic stochastic c-rescale pressure coupling.^31^

### Biodistribution Studies

For biodistribution studies, immunocompetent B6 White C57BL/6N.Cg-Tyr c/Rj mice with a C57BL genetic background (Janvier Laboratories, Le Genest-Saint-Isle, France) were injected in the tail vein with G3-CYS-Cy7.5 (1, 5, 10, or 20 mg/kg) and fluorescence recorded using an IVIS system (Revvity, Waltham, MA; USA) and fluorescence recorded at the indicated times. Cy7.5 fluorescent signals were recorded using a filter pair of 745 nm of excitation and 820 nm for emission. The experiments were conducted in accordance with the procedure approved by the Experimental Animal Committee of the Universidad de Castilla-La Mancha (permit code PR-2023–14).

### Coagulation Studies

Coagulation studies were performed as previously described.^32^ Blood was obtained by intracardiac extraction from B6 mice and centrifuged immediately to remove red blood cells and buffy coats, yielding plasma. The procedure was reviewed and approved by the Experimental Animal Committee of the Universidad de Castilla-La Mancha (permit code PR-2023–14).

Nanoparticle solutions (1 μM and 5 μM) were prepared in 0.9% (w/v) NaCl and added to the plasma in a 96-well plate in duplicate. Coagulation was initiated by adding CaCl_2_ to a final concentration of 20 mM to each well, except for the controls, and clotting was monitored every minute for 50 min at 405 nm using a Victor3 1420 spectrophotometer (PerkinElmer, Madrid, Spain).

Results were expressed as

%clotting=((Abs sample−Absblank))/((Abs C−Absblank))×100

Statistical analysis: The nonparametric variance analysis (Kruskal–Wallis) followed by Dunn’s test was used to evaluate statistical differences between groups. *p* < 0.05 was considered statistically significant. Statistical analyses were performed using GraphPad software (GraphPad Software; Boston, USA).

## Results and Discussion

### Synthesis of Cationic Bis-MPA Dendrimers Functionalized with Cysteamine or Dimethylamine Hydrochloride for siRNA Delivery

Building upon previous studies demonstrating the ability of β-alanine-functionalized bis-MPA dendrimers to complex siRNA, protect it from enzymatic degradation, and maintain low cytotoxicity,^14^ we aimed to further optimize these vectors for improved transfection performance. While promising, the transfection efficiency of β-alanine dendrimers remained modest, potentially due to suboptimal interaction with siRNA, which may hinder complex stability, or rapid intracellular degradation leading to premature siRNA release. To investigate the role of hydrolytic stability in transfection outcomes, we synthesized a series of bis-MPA dendrimers spanning the first to third dendritic generations, functionalized with either cysteamine or dimethylamine hydrochloride—two functionalities previously shown to significantly enhance hydrolytic stability^15^ (Figure 1). For clarity, these dendrimers (TMP-Gn-[CYS]*_m_* and TMP-Gn-[DA]*_m_*, where *n* = 1–3 and *m* = 6–24) are referred to herein as Gn-CYS/DA, where “*n*” represents the dendritic generation (*n* = 1–3), and “CYS” or “DA” indicate the peripheral functionality with cysteamine or dimethylamine HCl, respectively, while maintaining the same bis-MPA dendritic core (TMP).

**Figure 1:**
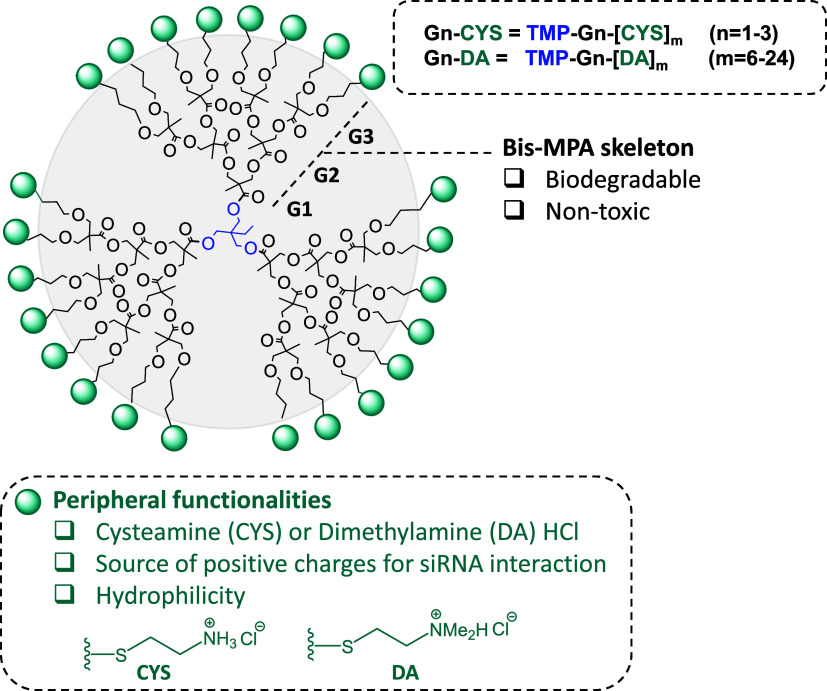
Structures of the cationic bis-MPA based dendritic polymers included in this study. Bis-MPA dendrimers functionalized with cysteamine HCl (CYS) and 2-(Dimethylamino)­ethanethiol hydrochloride (DA) as potential vectors of siRNA.

### Dendrimer-siRNA Interaction and Protection from RNases

To serve as effective siRNA carriers *in vivo*, dendrimers must not only bind siRNA but also protect it from RNase-mediated degradation, ensuring that intact siRNA can circulate through the bloodstream and reach the target tissue to knock down the intended protein.^19^ To study siRNA binding, we performed gel retardation assays as described in the Experimental Section.

As shown in Figure 2, the G1 dendrimer functionalized with 2-(dimethylamino)­ethanethiol (G1-DA) failed to complex more than 50% of 100 nM siRNA, even at a N/P ratio of 14.28, excluding it from further evaluation. In contrast, higher-generation DA-functionalized dendrimers effectively complexed 100 nM siRNA, with G3-DA demonstrating superior potency (N/P ratio: 2.85) compared to G2-DA (N/P ratio: 14.28), consistent with the increased number of surface-positive charges in the third generation (Figure 2). Cysteamine (CYS)-functionalized bis-MPA dendrimers also bound siRNA effectively, although with varying efficiencies. G1-CYS achieved full complexation at a N/P ratio of 14.28, while both G2-CYS and G3-CYS required only N/P ratios of 1.42 and 2.85, respectively.

**Figure 2:**
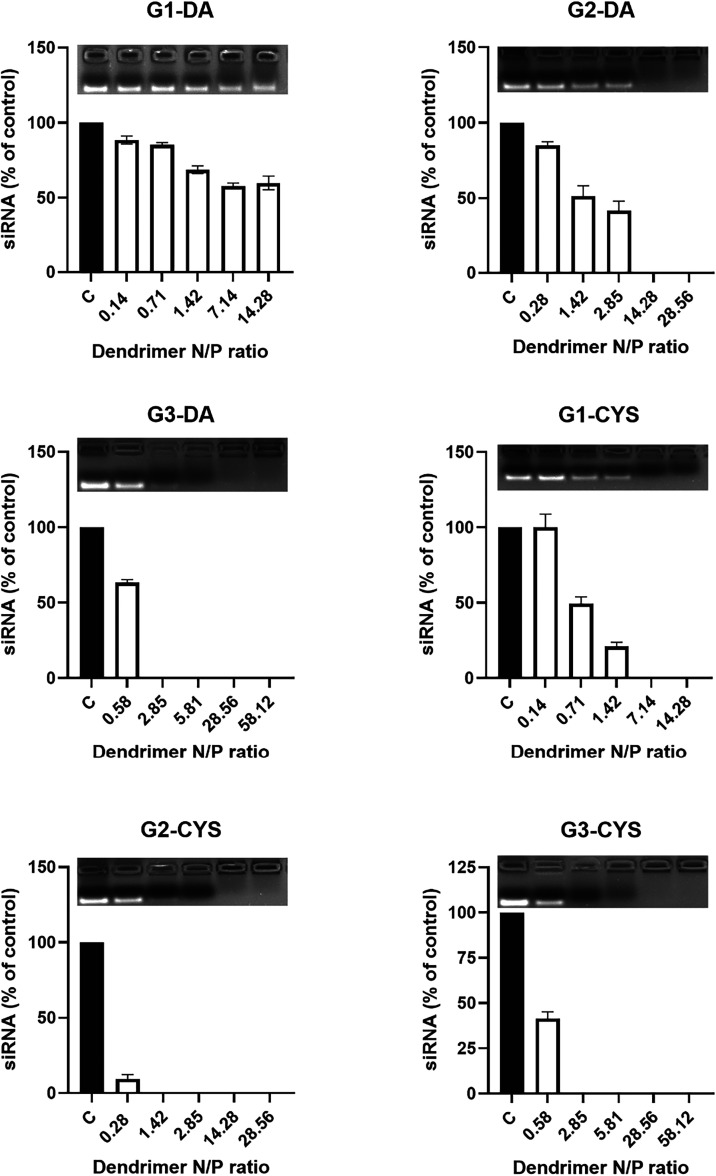
Gel retardation studies. siRNA (100 nM) was incubated with G1–G3 DA- or CYS-terminated dendrimers at increasing N/P ratios, and unbound siRNA was quantified as described in the Experimental Procedures. Representative gels are shown above each graph. Data represent mean ± SEM (*n* = 3–4).

These findings highlight two critical factors influencing siRNA complexation: dendrimer generation and the chemical nature of the terminal functional groups including the nature of terminal amines, two factors that shed light for future design of siRNA-transfecting nanoparticles. Regarding dendritic generation, at least 12 surface-positive charges (as in G2) appear necessary for efficient siRNA binding. Within the family of cysteamine functionalized bis-MPA dendrimers, it is interesting to note that G2-CYS demonstrated greater complexation efficiency than G3-CYS, suggesting that an excessive density of surface-positive charges may hinder accessibility or create steric hindrance.

The nature of the peripheral amine also plays a pivotal role. Despite having an equal number of positive charges, G2-DA was significantly less potent than G2-CYS. This discrepancy likely arises from the fundamental chemical differences between the amines. Protonated primary amines, such as those in cysteamine, possess a more localized and concentrated positive charge, enhancing electrostatic interactions with the negatively charged siRNA. In contrast, the dimethylated nitrogen in DA groups experiences electron donation from the methyl substituents, which slightly diminishes its positive charge density. Additionally, the bulky dimethylamine group may sterically hinder interactions between the RNA bases and the surrounding positive charge. Although protonated tertiary amines can also interact with siRNA bases, primary amines are capable of forming stronger and more directional hydrogen bonds due to their less hindered geometry and multiple N–H donors, likely contributing to the enhanced stability observed in these complexes.

Next, we investigated the ability of the dendrimers to protect siRNA from RNase-mediated degradation, a critical requirement for their potential biomedical application as siRNA carriers. Overall, all dendrimers protected siRNA from degradation, although with different efficacy, which increased with dendrimer generation (Figure 3). Specifically, G1-CYS protected approximately 35% of siRNA, while both second-generation dendrimers (G2-CYS and G2-DA) offered around 50% protection. Third-generation dendrimers (G3-CYS and G3-DA) demonstrated the highest efficacy, protecting up to 75% of siRNA. This trend is consistent with the increasing number of surface-positive charges in higher-generation dendrimers, which likely enables more complete siRNA coverage. This, in turn, would reduce the Solvent-Accessible Surface Area (SASA) of the siRNA, thereby limiting RNase binding and subsequent degradation.^33^ However, the higher p*K*_a_ of protonated tertiary amines, which may reduce their efficacy in siRNA delivery, might not be the only factor contributing to the observed differences in dendrimer–siRNA interactions. Steric hindrance caused by the bulkier dimethylamine groups could also play a significant role by limiting close interactions with RNA bases and surrounding positive charges. Thus, the differences in dendrimer performance are likely due to a combination of factors, including p*K*_a_, steric effects, and potential hydrogen-bonding interactions.

**Figure 3:**
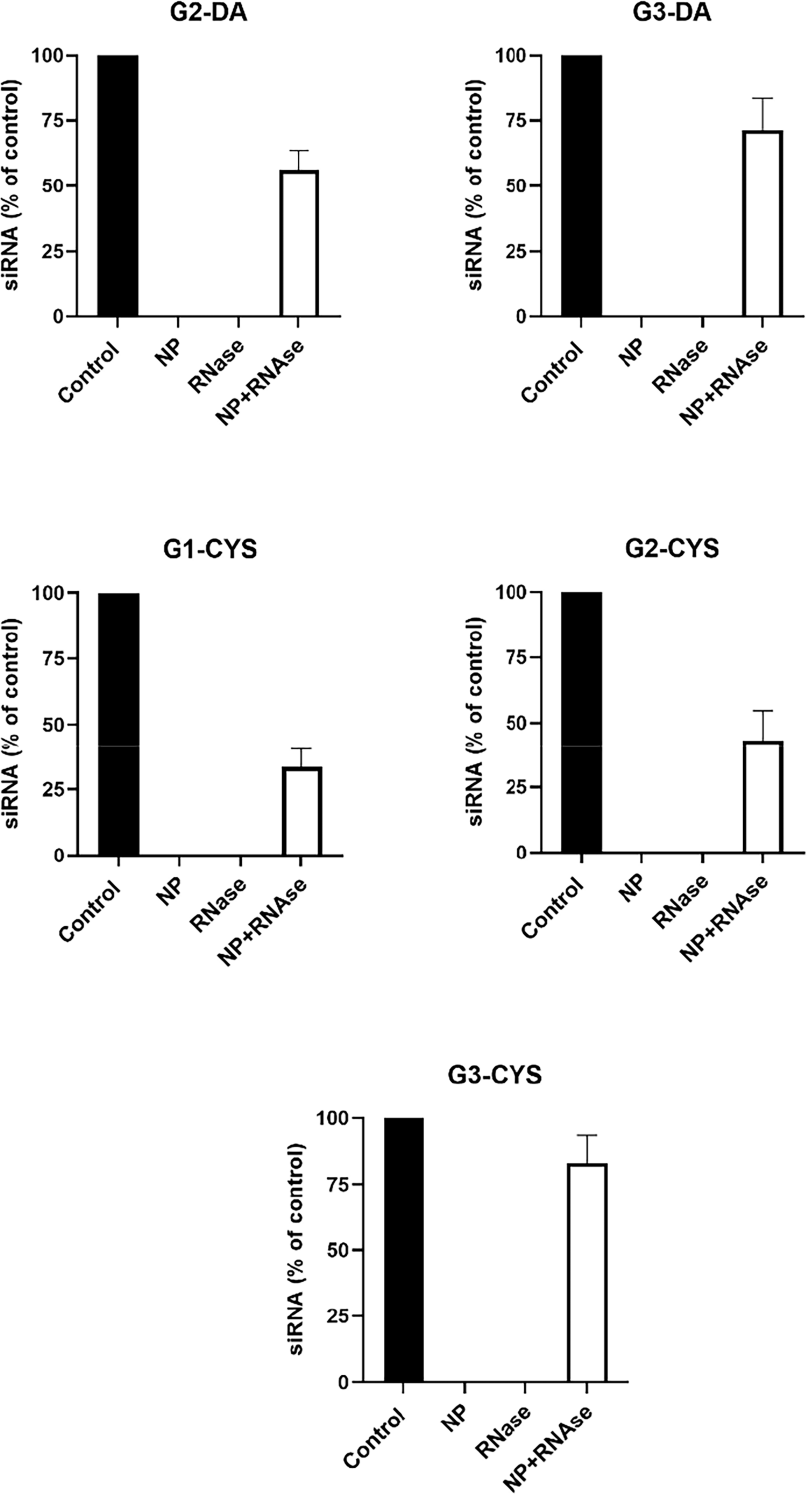
Dendrimer-mediated protection of siRNA against RNases. Dendrimers (G2-DA [5 μM], G3-DA [0.5 μM], G1-CYS [5 μM], G2-CYS [0.5 μM], and G3-CYS [0.5 μM]) were incubated with 100 nM siRNA, and the degree of protection was determined as described in the Experimental Procedures. Data are reported as mean values, with error bars indicating the SEM for *n* = 3–4.

### Cytotoxicity Assessment of Dendrimers

Confident in the dendrimers’ ability to effectively bind and protect siRNA from RNase-mediated degradation, the next step was to evaluate their capacity to knock down key proteins implicated in GBM cell proliferation, survival, and treatment resistance, including p42-MAPK, Rheb, and MGMT.^34^ To achieve this, we first evaluated the toxicity of the dendrimers in the commercial human GBM cell line T98G, with the goal of selecting the least toxic candidate for subsequent transfection and in vivo biodistribution studies. Toxicity was initially assessed using a lactate dehydrogenase (LDH) assay to measure cellular damage. Given that high toxicity in a commercial cell line at concentrations relevant to siRNA transfection would likely predict severe effects in neurons or astrocytes, this assay served as a critical preliminary screening step.

Figure 4 shows that after 72 h of exposure to increasing dendrimer concentrations, both G2-DA and G3-DA dendrimers exhibited a sharp increase in cellular death—by more than 10-fold—when the concentration was raised from 1 μM (G2) or 3 μM (G3) to 5 μM, representing a 5-fold or smaller increase in dose. Similarly, for the CYS-functionalized dendrimers, G1 did not induce any toxicity at concentrations up to 10 μM, whereas both G2 and G3 caused a pronounced rise in cell death at concentrations above 1 μM (Figure 4). The toxic effect was quite fast in cell cultures (Supporting Information Video). These toxic effects are likely attributable to the positive charges on the dendrimers interacting with negatively charged cell membranes, resulting in nanopore formation, blebs, membrane disruption, and subsequent cell death.^35^ Importantly, these cytotoxic effects occurred at concentrations only slightly above those selected for transfection in the RNase protection studies (0.5 to 1 μM). Although this indicates a narrow therapeutic window between transfection-effective and toxic concentrations, it still permits the assessment of dendrimer-mediated transfection efficiency.

**Figure 4:**
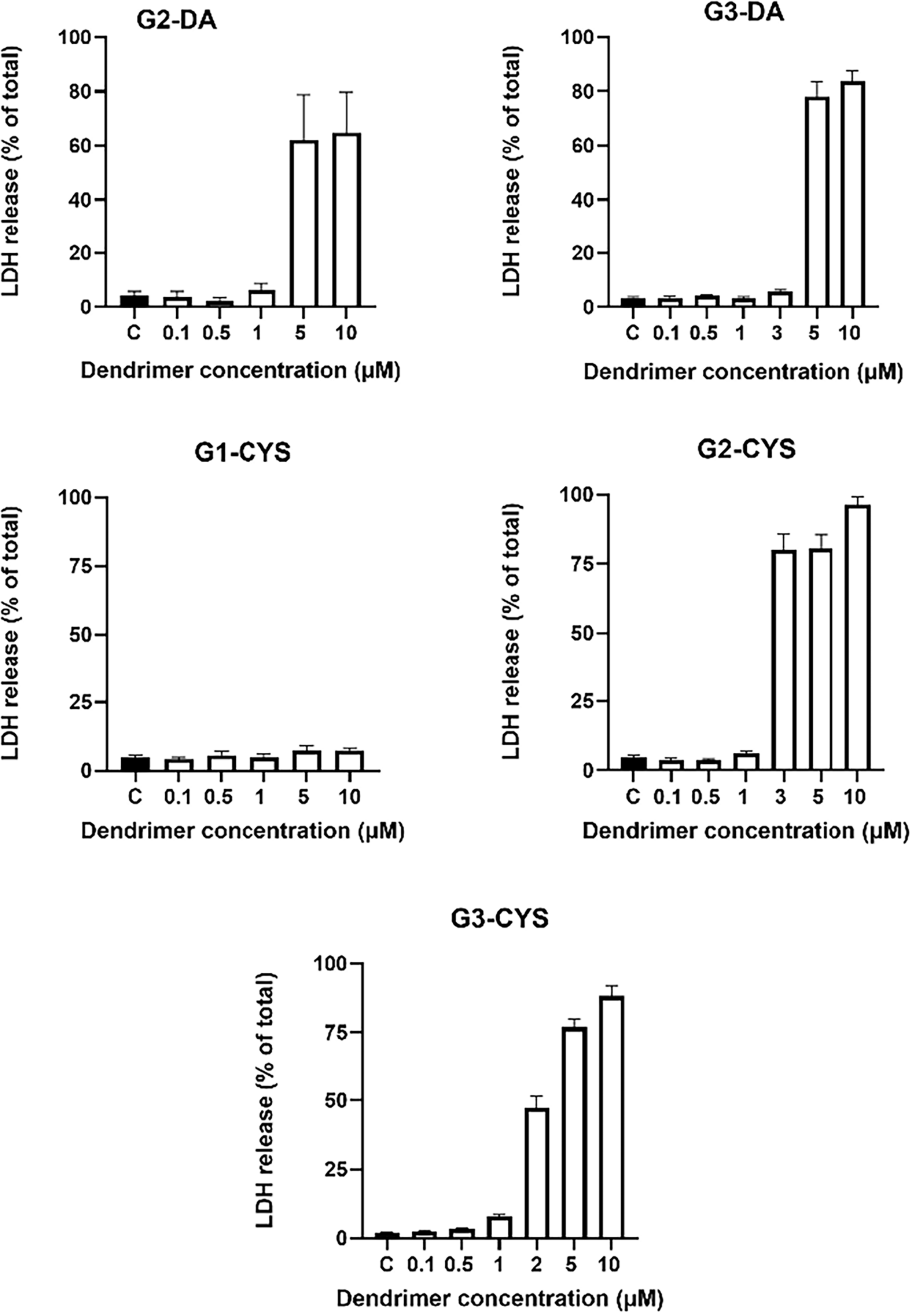
Direct toxicity of the dendrimers. T98G cells were exposed to increasing dendrimer concentrations ranging from 0.1 to 10 μM and toxicity determined as LDH release to the culture medium determined at 72 h. Data are reported as mean values, with error bars indicating the SEM for *n* = 8 to 24.

To compare the biological activity of Bis-MPA-based dendrimers with commercially available reagents, we examined the toxicity of three commonly used transfection agents: Lipofectamine RNAiMAX, Fugene, and G3-PAMAM dendrimers. While neither Lipofectamine RNAiMAX nor G3-PAMAM showed any toxicity toward T98G cells, even at concentrations 10-fold higher than those recommended for transfection (Supporting Information, Figure SI3), Fugene exhibited significant toxicity toward T98G glioblastoma (GBM) cells at concentrations above those recommended by the supplier for transfection studies.

Given that efficient siRNA transfection is often challenging to achieve, and that Bis-MPA dendrimers provided effective siRNA protection at concentrations below their cytotoxic threshold, we subsequently investigated the transfection efficiency of both families of Bis-MPA-based dendrimers.

### siRNA Delivery Efficiency into GBM Cells

After determining the optimal concentrations at which the dendrimers effectively bind siRNA and protect it from degradation without causing significant cytotoxicity, we evaluated their capacity to facilitate siRNA uptake into GBM cells. Dendriplexes were prepared by combining G2-DA (5 μM), G3-DA (0.5 μM), G1-CYS (5 μM), G2-CYS (0.5 μM), or G3-CYS (0.5 μM) with 100 nM 6-carboxyfluorescein (FAM)-labeled siRNA. The efficiency of siRNA delivery was assessed by quantifying intracellular fluorescence in T98G cells after 6 h of incubation.

As shown in Figure 5, siRNA delivery was most effective with G3-CYS dendriplexes, as evidenced by intense intracellular fluorescence. A weak signal was observed for G2-CYS, whereas no fluorescence was detected for DA-terminated dendrimers, indicating minimal or absent siRNA uptake. These results suggest that among the tested dendrimers, G3-CYS exhibited the highest delivery efficiency and is the most promising candidate for siRNA-mediated knockdown of target proteins in GBM cells. Overall, these findings support the further investigation of G3-CYS dendrimers as potential vehicles for siRNA-based gene silencing in GBM therapy.

**Figure 5:**
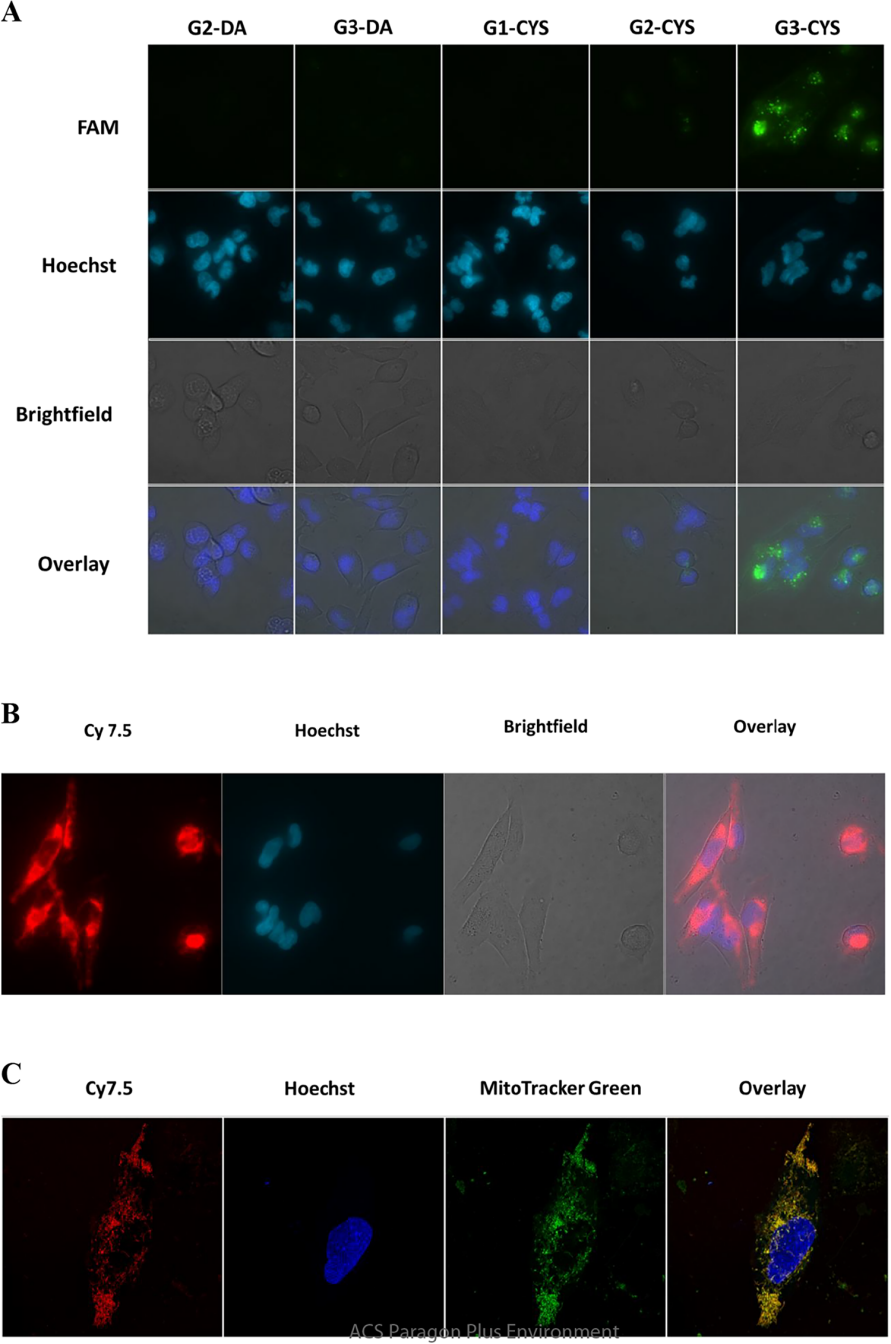
Cellular uptake of dendrimer/siRNA dendriplexes in T98G cells. (A) T98G cells were incubated for 6 h with dendriplexes formed by FAM-labeled siRNA (100 nM) and dendrimers at the following concentrations: G2-DA (5 μM), G3-DA (0.5 μM), G1-CYS (5 μM), G2-CYS (0.5 μM), or G3-CYS (0.5 μM). Shown are siRNA (FAM), nuclei (Hoechst), brightfield, and merged images. The experiment was performed twice with similar results. (B) Cells were incubated for 6 h with Cy7.5-labeled G3-CYS (1 μM) and Hoechst. Brightfield and merged images are also shown. The experiment was performed twice, with eight frames recorded each time, yielding similar results. (C) Cells were incubated for 6 h with Cy7.5-labeled G3-CYS (1 μM) and confocal images recorded. The figure shows a confocal image of a T98G cell at 2.4 μm above the basal plane, showing intracellular Cy7.5-labeled G3-CYS dendrimers localized in the same plane as mitochondria. Shown are Cy7.5, nuclei (Hoechst), mitochondria (MitoTracker Green), and merged images. The experiment was performed four times with similar results.

Cy7.5-labeled G3-CYS was incorporated into nearly 100% of T98G cells, demonstrating that, consistent with its ability to deliver siRNA and mediate target protein knockdown, the dendrimer itself efficiently entered GBM cells (Figure 5B). To verify that the observed dendrimer-associated fluorescence was intracellular rather than surface-bound, we performed confocal microscopy. This analysis confirmed intracellular localization of the Cy7.5-labeled dendrimer, as the fluorescence signal was detected within the same focal plane as the mitochondria (Figure 5C).

The limited siRNA transport into T98G GBM cells observed for most dendrimers may be attributed to two primary factors. First, the chemical composition of the dendrimer surface plays a critical role. Dendrimers functionalized with dimethylamine (DA) groups were significantly less effective in mediating cellular uptake compared to those bearing cysteamine (CYS) groups. This observation is consistent with previous findings in siRNA delivery, where a series of cationic shell-cross-linked nanoparticles with varying ratios of primary to tertiary amines demonstrated that the formulation containing only primary amines achieved the highest silencing efficiency in HeLa cells. The enhanced silencing was attributed to improved cellular uptake, highlighting the critical role of primary amines in facilitating efficient transfection.^36^

Second, the dendrimer generation influences the number of protonatable surface charges, which are crucial for effective siRNA binding and cellular transport. Additionally, the hydrophobic–hydrophilic balance, influenced by the dendrimer’s structural size, may further affect delivery efficiency. The polyester-based dendritic core introduces hydrophobic character, while the peripheral cationic groups contribute to hydrophilicity. Lower-generation dendrimers (G1-CYS and G2-CYS), with fewer surface charges, either fail to mediate siRNA transport or result in only weak intracellular fluorescence signals. In contrast, G3-CYS/siRNA dendriplexes produce robust intracellular fluorescence, indicating superior delivery efficiency. These findings suggest that, beyond the chemical identity of the surface groups (e.g., cysteamine), a critical threshold of positive charges is required for efficient siRNA delivery.^37^

### siRNA Transfection Efficiency

While siRNA uptake experiments narrowed the pool of promising dendrimers for transfection to G3-CYS, we proceeded to evaluate the transfection efficiency of the entire cysteamine-functionalized dendrimer family, along with G2-DA and G3-DA dendrimers. As anticipated based on the uptake results (Figure 5), neither G2-DA nor G3-DA reduced intracellular levels of p42-MAPK or Rheb in T98G cells (Figure SI1), consistent with their poor siRNA delivery capabilities. Similarly, G1-CYS and G2-CYS failed to lower target protein expression (Figure SI2), suggesting that the limited siRNA uptake observed for G2-CYS (Figure 5) was insufficient to elicit gene silencing.

In contrast, G3-CYS effectively transported siRNA into T98G cells and reduced the intracellular levels of p42-MAPK, Rheb, and MGMT to 5–20% of control, confirming potent target knockdown (Figure 6). This highlights the importance of the high density of positive charges^38^ contributed by the cysteamine terminal groups—24 in G3-CYS—which confer excellent transfection performance. Encouraged by the strong efficacy of G3-CYS in the T98G human GBM cell line, we next investigated whether it could also achieve efficient siRNA transfection in primary cell cultures, which are typically far more resistant to transfection. Accordingly, we extended our studies to siRNA transfection in primary astrocyte cultures.

**Figure 6:**
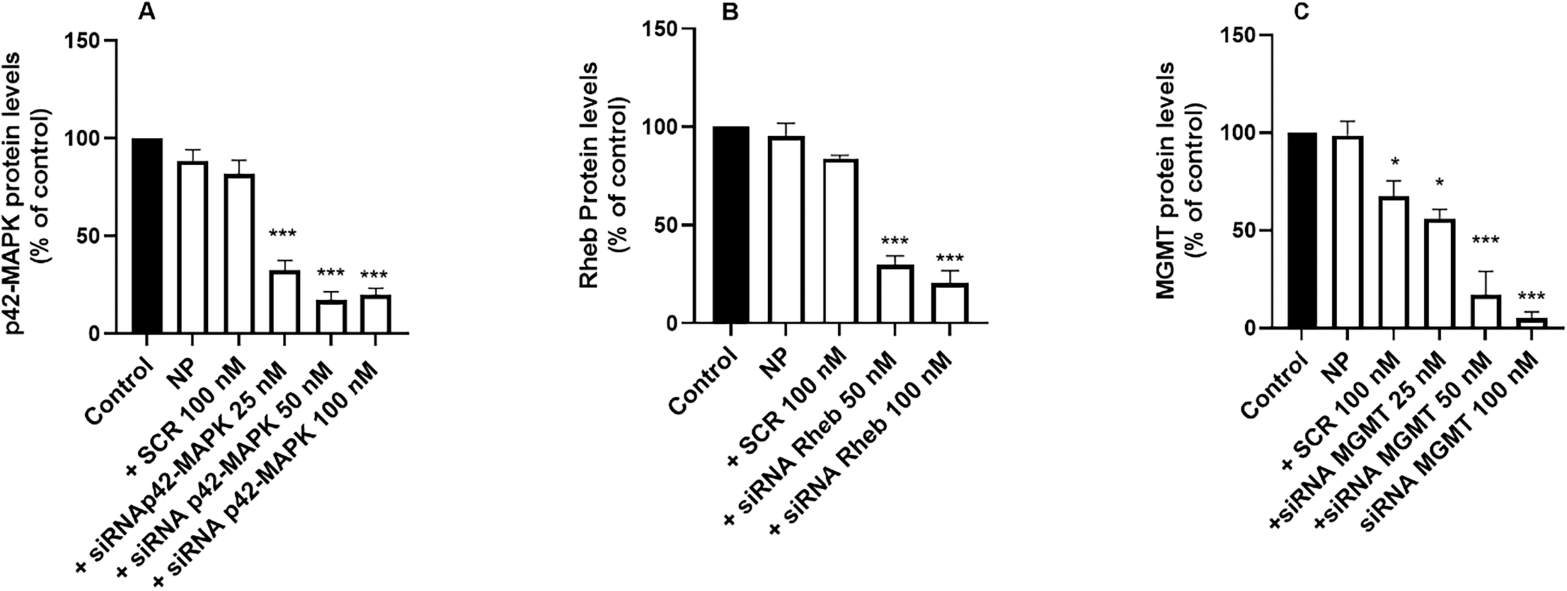
Knockdown of p42-MAPK, Rheb, and MGMT proteins in T98G GBM cells. Dendriplexes formed by G3-CYS (1 μM) and different concentrations of siRNA (25 to 100 nM) targeting either (A) p42-MAPK, (B) Rheb, or (C) MGMT were incubated with T98G cells for 72 h. Cellular content was then quantified as described in the Experimental Procedures section. Data represent the mean, with error bars indicating the SEM of 4 to 6 experiments. *p < 0.05; *p < 0.001.

Interestingly, as shown in Figure 7, siRNA delivered by G3-CYS significantly reduced p42-MAPK protein levels to approximately 30% of control values and lowered Rheb levels to about 15% of control. However, no transfection was detected in primary neuronal cultures (data not shown), suggesting that further structural optimization of the dendrimer may be necessary to achieve efficient siRNA delivery in neurons.

**Figure 7:**
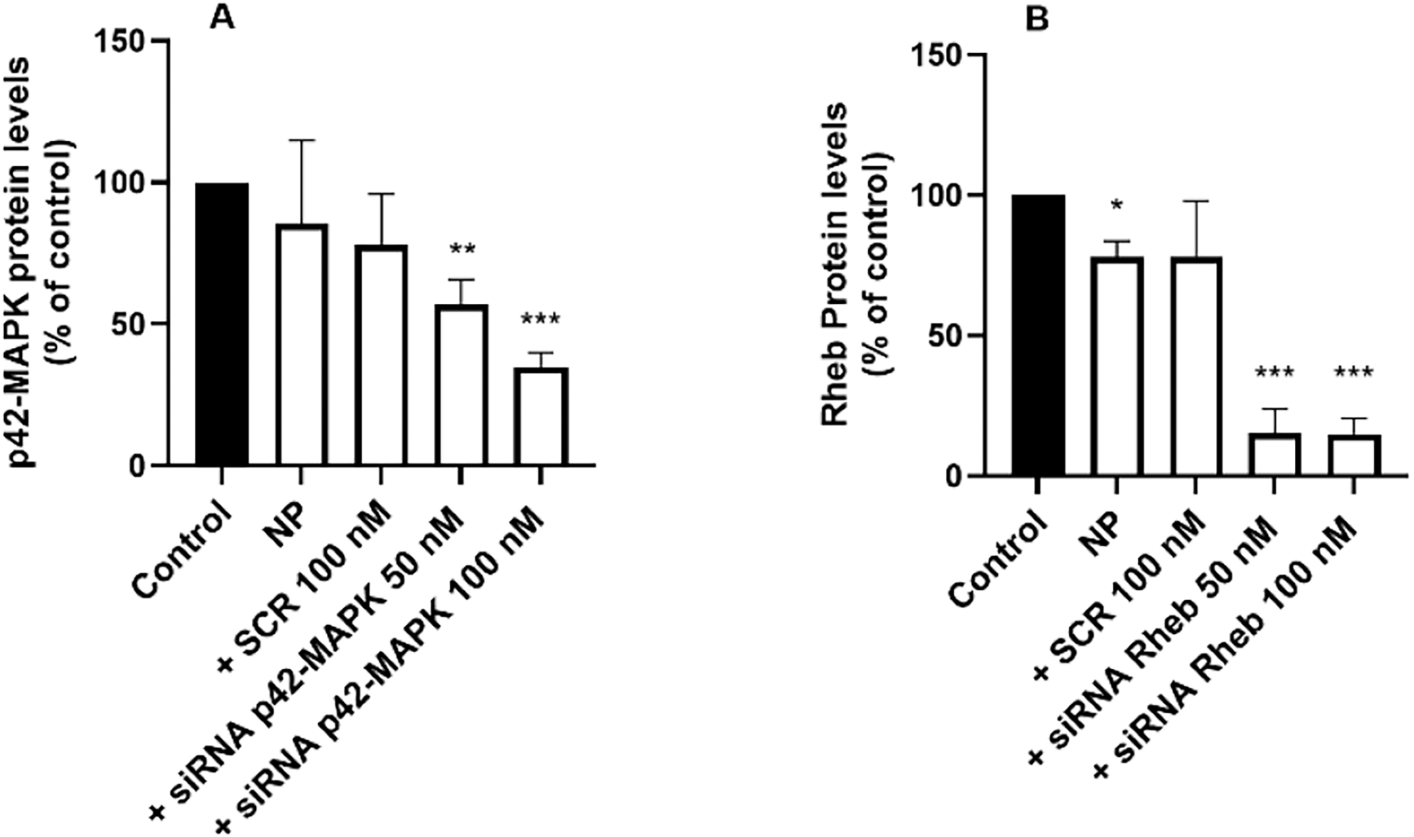
Knockdown of p42-MAPK and Rheb proteins in mouse primary astrocytes. Dendriplexes were formed by incubating G3-CYS (1 μM) and different concentrations of either scramble siRNA (SCR) or siRNA (50 or 100 nM) targeting either (A) p42-MAPK or (B) Rheb. Then the dendriplexes were incubated with primary astrocytes for 72 h. Cellular protein content was then quantified as described in the Experimental Procedures section. Data represent the mean with error bars indicating the SEM of 4 to 6 experiments. *p < 0.05; **p < 0.01; ***p < 0.001 when compared to control.

To evaluate the transfection efficiency of G3-CYS relative to commonly employed transfection agents, we investigated the transfection performance of Lipofectamine RNAiMAX, Fugene, and PAMAM G3 in human T98G glioblastoma cells and primary mouse astrocytes. The findings indicate that G3-CYS exhibits transfection efficiencies comparable to those of Lipofectamine RNAiMAX and Fugene, whereas PAMAM G3 failed to achieve transfection in either T98G cells or mouse astrocytes (Supporting Information, Figures SI4 and SI5). Importantly, G3-CYS offers a potential advantage over conventional reagents, as its peripheral groups can be readily functionalized, thereby enabling additional applications such as targeted delivery and imaging probe conjugation.

### *In Vivo* Biodistribution

The promising siRNA transfection activity observed in vitro with the G3-CYS dendrimer prompted us to investigate its in vivo performance. As a first step, we assessed the biodistribution of the dendrimer in mice to determine whether it could reach the central nervous system. To this end, the dendrimer was labeled with NHS-activated cyanine 7.5 (Cy7.5; Figure 8A). Cy7.5 is a near-infrared dye commonly used to label biomolecules, including proteins, due to its low tissue absorption, minimal scattering, and high signal-to-noise ratio—properties that make it well-suited for in vivo imaging applications.^39^

**Figure 8:**
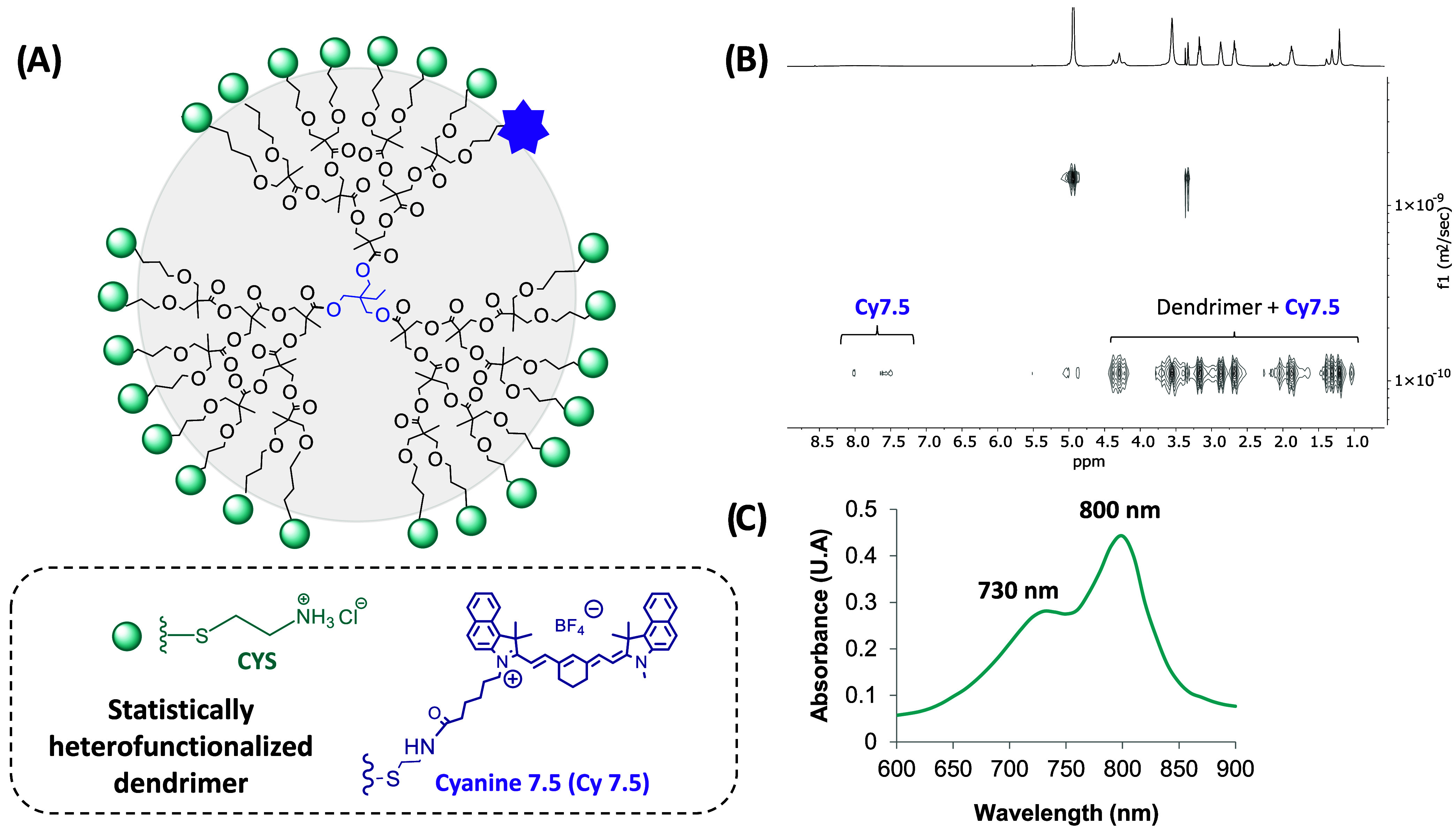
(A) Proposed structure for the third generation cationic bis-MPA dendrimer functionalized with Cy7.5 (G3-CYS-Cy7.5), (B) DOSY 2D NMR in CD_3_OD, (C) UV–vis spectrum for the labeled dendrimer in DI water at 10 μM.

A 1:1 molar ratio of dendrimer to dye was used to statistically label a single dendritic branch. Due to the limited water solubility of the dye, the reaction was carried out in a DI water/DMSO mixture (1:0.3 v/v). After stirring the reaction mixture overnight at 50 °C in the dark, the product was dialyzed to remove any unbound dye. The labeled dendrimer was obtained as a green solid in high yield (85%). Successful conjugation of Cy7.5 was confirmed using ^1^H NMR, DOSY-2D, and UV–vis spectroscopy. As shown in Figure 8B, the aromatic signals of the dye (7.0–8.0 ppm) in the 1H NMR spectrum shared the same diffusion coefficient as the dendrimer in DOSY analysis, indicating successful attachment and the absence of free dye. Furthermore, UV–vis spectroscopy confirmed that dye conjugation did not alter its absorption profile, with the labeled dendrimer exhibiting a maximum absorbance near 800 nm (Figure 8C), consistent with the free dye.^40^

For biodistribution studies, we began by intravenously (i.v.) injecting 20 mg/kg of G3-CYS-Cy7.5, a standard dose in our laboratory, that typically yields a strong fluorescent signal. However, shortly after injection (approximately 1 h), all animals died. We subsequently reduced the dose to 10 mg/kg and then to 5 mg/kg, but the toxicity remained comparable to that observed at 20 mg/kg. A further reduction to 1 mg/kg allowed the animals to survive; however, no fluorescent signal was detected (data not shown), suggesting that the total amount of nanoparticle administered was below the detection threshold of the imaging equipment.

To correlate these findings with the in vitro toxicity data, we estimated the plasma concentration of G3-CYS-Cy7.5 following i.v. injection of the 20 mg/kg dose (500 μg for a 25 g mouse). Assuming a total blood volume in the mouse of 1.8 mL,^41^ we calculated an approximate plasma concentration of 30 μM while at 5 mg/kg dose the estimated plasma concentration was about 7.5 μM. In both cases, the concentrations exceeded 3 μM, which induced >70% mortality in GBM T98G cells in vitro (Figure 4, G3-CYS). This high systemic concentration likely accounts for the rapid death of the animals.

To further investigate the mechanism underlying this acute toxicity at dendrimer concentrations above 1 μM, we recorded the cellular response to G3-CYS (10 μM) in T98G cultures. As shown in the time-lapse video (Supporting Information Video), within 1 min of G3-CYS addition, cells began to lose their defined borders. By 3 min, membrane blebbing was evident, progressing to extensive blebbing and cell death by 5 min (note: the recording is shown at 32× speed). Amine-terminated polymers, such as G3-CYS, are known to interact with cell membranes, increasing permeability and inducing cytotoxicity.^42^ Furthermore, release of cytosolic enzymes like LDH is associated with increased membrane permeability, likely due to nanoscale pore formation or localized alterations in membrane composition (proteins, cholesterol, and lipids) caused by dendrimer–lipid interactions.^35^ These findings provide a mechanistic explanation for the high cellular toxicity of G3-CYS at concentrations above 1 μM, and for the death of animals at doses of 5, 10, and 20 mg/kg—all of which produce plasma concentrations exceeding 5 μM—likely due to cumulative membrane damage across multiple cell types.

Moreover, we studied the effect of the dendrimer on coagulation. As shown in Figure 9, a slight increase in calcium-induced coagulation of pooled serum from B6 mice was observed for the dendrimer at 1 μM. However, at a dendrimer concentration of 5 μM, serum coagulability increased significantly (Figure 9), which could also contribute to the observed mortality in the biodistribution experiments.

**Figure 9:**
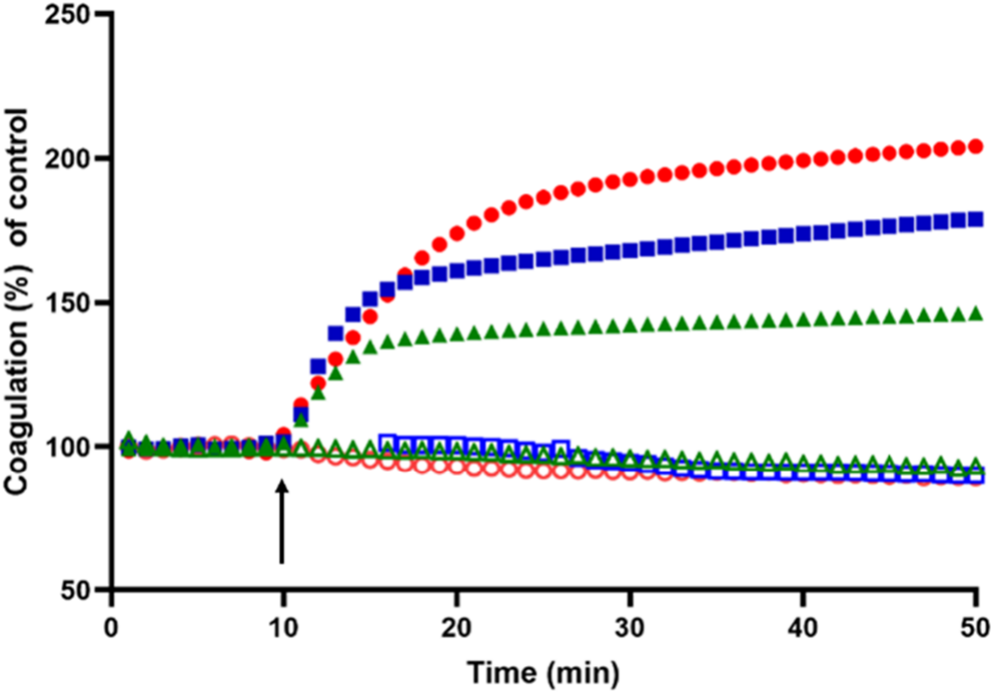
Calcium-induced coagulation in the absence and presence of G3-CYS. Coagulation levels were recorded every minute for 50 min and initiated at 10 min by the addition of Ca^2+^ (arrow). The graph shows normalized coagulation values under the following conditions: (a) In the absence of calcium: No nanoparticle (green open triangles); G3-CYS, 1 μM (blue open squares); G3-CYS, 5 μM (red open circles); (b) in the presence of calcium: No nanoparticle (green filled triangles); G3-CYS, 1 μM (blue filled squares); G3-CYS, 5 μM (red filled circles). Data represent the mean of four independent experiments. Error bars have been omitted for clarity.

### Molecular Dynamics and Particle-Size Evaluation

The intriguing biological behavior of G3-CYS dendrimers, particularly the sharp increase in toxicity with only slight changes in concentration, prompted us to further investigate their physicochemical properties in aqueous environments. To begin, we performed molecular dynamics (MD) simulations to explore the potential intermolecular interactions between dendrimers in solution. Given the high density of peripheral positive charges in cysteamine-functionalized polyester dendrimers, we hypothesized that strong electrostatic repulsion would occur between them.

Indeed, MD simulation results supported this hypothesis. Two 35 ns MD simulations of a pair of solvated G3-CYS dendrimers were conducted: one starting with the dendrimers well-separated and the other from an aggregated state (see Experimental Section and Figure 10A,B). In the first case, the dendrimers remained apart throughout the simulation, while in the second, the initial aggregate dissociated within 10 ns (Figure 10C). By the end of both simulations, no significant differences were observed between the two trajectories, indicating a strong repulsive interaction between the dendrimers under these conditions.

**Figure 10:**
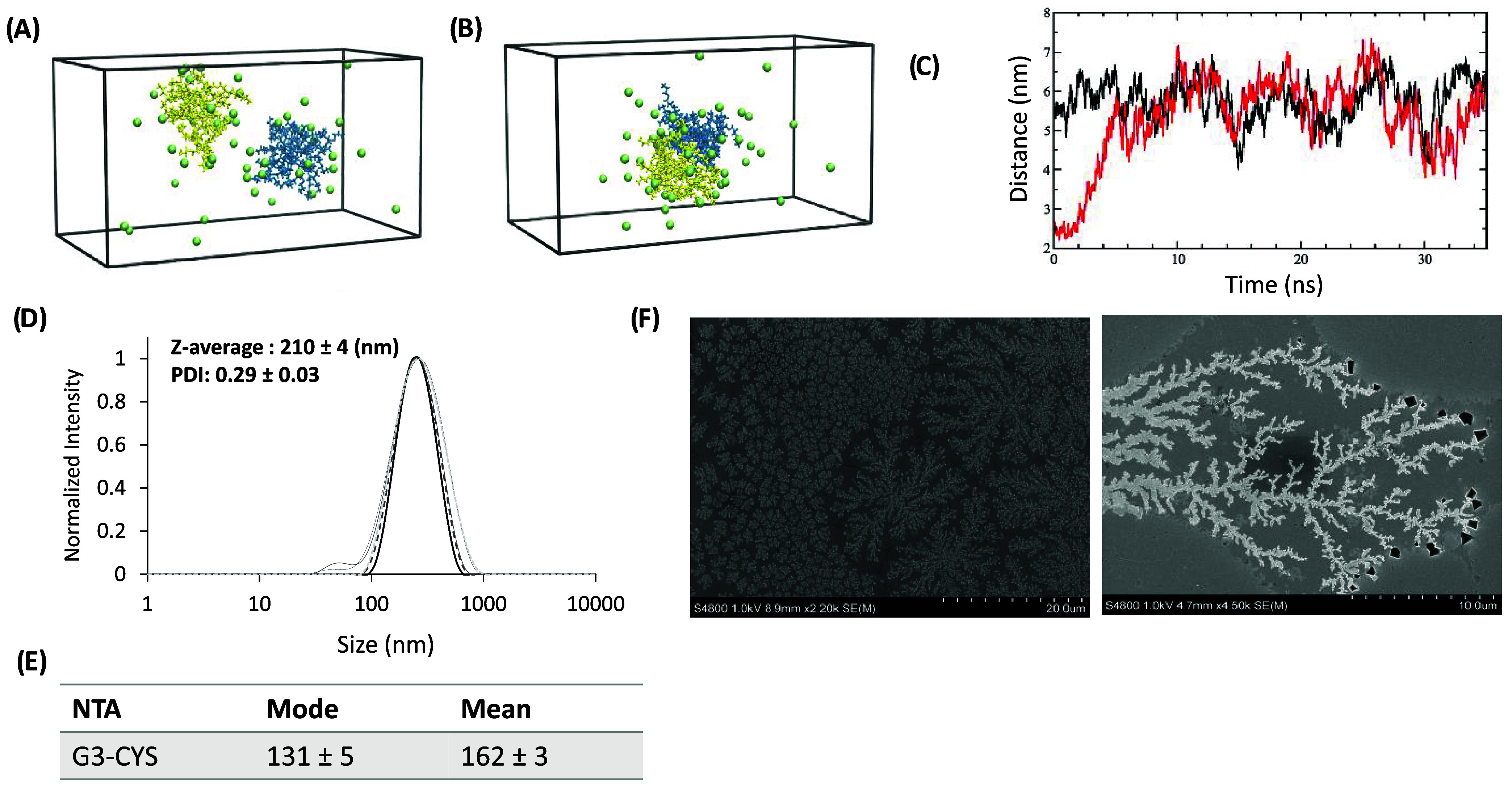
Size analysis for the dendrimer of third generation G3-CYS. Molecular dynamics (A, B, C): representative snapshots of the model system in (A) the separated and (B) aggregated states, respectively. The two dendrimers are colored yellow and blue, respectively, and chloride ions are green, (water molecules are not shown). (C) The distance between the centers of the two dendrimers as a function of time extracted from the simulations starting in the aggregated (red) and the separated (black) state. (D) Normalized intensity of the different populations measured in nanometers by DLS. (E) Mode and mean values (in nm) from NTA determinations. (F) SEM pictures of the dendrimer at 2.2 k (right) and 4.5 k (left) magnifications.

To experimentally assess the aggregation behavior of G3-CYS, we analyzed their size in both solid-state and aqueous environments using scanning electron microscopy (SEM), dynamic light scattering (DLS), and nanoparticle tracking analysis (NTA). Surprisingly, contrary to MD predictions, G3-CYS dendrimers aggregated under all tested conditions. In the solid state, SEM analysis revealed the formation of fractal-like aggregates upon drying aqueous dendrimer solutions (Figure 10F). To our knowledge, such aggregation has previously only been reported between oppositely charged dendrimers (e.g., carboxylate and amine-terminated PAMAM dendrimers),^43^ but not for systems with uniformly charged peripheries like G3-CYS.

Aggregation was also evident in solution. DLS and NTA, which both infer hydrodynamic diameter from Brownian motion, confirmed the presence of nanoscale aggregates. While DLS measures time-dependent fluctuations in scattering intensity, NTA tracks the movement of individual particles via video microscopy. DLS measurements in aqueous solution at 25 °C, comparable to the simulation conditions, revealed aggregates with an average size of approximately 210 nm and a polydispersity index of 0.3. A key distinction between the simulation and DLS experiments is the concentration: DLS requires relatively high sample concentrations (∼500 μM), whereas the MD simulation involved only two molecules, potentially explaining the contrasting results.

To better mimic physiological conditions, NTA was performed at 37 °C in phosphate-buffered saline (PBS). The dendrimer showed an average hydrodynamic diameter of 162 nm, with the majority of particles around 131 nm in size. These results further support the unexpected aggregation behavior of G3-CYS dendrimers in solution. Rather than existing as discrete nanostructures, they form larger aggregates with increased surface-positive charge density.

Although the aggregation of cationic dendrimers is not widely documented, a related study by Kurokawa et al. reported low blood-brain barrier permeability of protonated amine-terminated PAMAM dendrimers, attributed to their aggregation upon contact with biological fluids.^44^ Despite the extensive literature on cationic polymers, the role of aggregation in their biological activity remains poorly understood. We propose that such aggregation increases the local density of surface-positive charges, thereby enhancing interactions with negatively charged cell membranes. This, in turn, may potentiate membrane disruption and toxicity at both cellular and systemic levels, as previously suggested by studies on polymer–lipid interactions.^35^

## Conclusions

Cysteamine-terminated bis-MPA dendrimers demonstrate superior siRNA binding affinity compared to their 2-(dimethylamino)­ethanethiol-functionalized analogs, underscoring the importance of surface chemistry and charge density in nucleic acid complexation. Among the tested constructs, the third-generation G3-CYS dendrimer exhibited the most effective siRNA complexation and cellular delivery capabilities. Fluorescence imaging confirmed efficient intracellular delivery of FAM-labeled siRNA into T98G human glioblastoma (GBM) cells exclusively with G3-CYS, correlating with a marked reduction to 15–25% of control levels in target protein expression of p42-MAPK, Rheb, and MGMT. Notably, G3-CYS also achieved robust siRNA transfection in primary mouse astrocytes, a cell type typically resistant to nonviral delivery methods, indicating its potential utility in difficult-to-transfect primary neural cell types. G3-CYS showed transfection efficiency similar to both Fugene and Lipofectamine RNAiMAX, two widely used commercial transfection reagents. However, it also presents two important advantages over them: first, because it is based on a bis-MPA backbone, it is self-degradable, which prevents dendrimer accumulation inside cells or organisms—something that generally does not occur with other dendrimers. Second, its peripheral groups can be readily functionalized, enabling additional applications such as targeted delivery and imaging probe conjugation, which are not feasible with either Fugene or Lipofectamine RNAiMAX. This represents a significant advance over other transfectants since G3-CYS combines excellent siRNA transfection properties with a self-degradable backbone which prevents accumulation inside the target cells and organs.

A steep concentration-dependent increase in cytotoxicity was observed above 1 μM, coinciding with the effective transfection range. Live-cell imaging and LDH release assays suggested that toxicity results from rapid dendrimer-induced membrane perturbation, likely through nanoscale pore formation or disruption of membrane integrity. Moreover, G3-CYS, at concentrations slightly below the estimated plasma levels achieved after intravenous administration, markedly increases plasma coagulability, which could contribute to the observed in vivo toxicity by producing vascular thrombosis. Contrary to molecular dynamics simulations, which predicted strong electrostatic repulsion between highly charged dendrimers, experimental data from dynamic light scattering (DLS), nanoparticle tracking analysis (NTA), and scanning electron microscopy (SEM) revealed that G3-CYS forms aggregates both in aqueous solution and in the solid state. This aggregation behavior may lead to locally enhanced surface charge densities, contributing to both increased membrane interaction and cellular toxicity. These findings establish G3-CYS as a potent and efficient siRNA delivery vehicle for glioblastoma cells and primary astrocytes, with significant implications for RNAi-based therapeutic strategies, while emphasizing the importance of precise dose optimization to balance structural design, biological efficacy, and safety.

## Supplementary Material





## Notes

Synthetic protocol for Cy7.5 labeling of G3-CYS dendrimer; T98G transfection by G2 and G3-DA dendrimers; T98G transfection by G1 and G2-CYS dendrimers; toxicity of Fugene, Lipofectamine RNAiMAX, and PAMAM G3 on T98G cells; T98G transfection by Fugene, Lipofectamine RNAiMAX, and PAMAM G3 (PDF)
Video showing cell toxicity in culture (MP4)

## Abbreviations

ATCC: American Type Culture Collection
bis-MPA: bis­(hydroxymethyl)­propionic acid
BSE: Backscattered Electron detector
CHARMM: Chemistry at Harvard Macromolecular Mechanics
Cy7.5: cyanine7.5 NHS ester
CYS: cysteamine
DA: 2-(dimethylamino)­ethanethiol
Dl wáter: deionized water
DMEM: Dulbecco’s modified Eagle’s medium
EDS: energy dispersive X-ray spectrometry
DLS: dynamic light scattering
DMSO: dimethyl sulfoxide
DOSY: diffusion ordered spectroscopy
EDTA: ethylenediamine tetra-acetic acid
Erk2: extracellular signal-regulated kinase 2
FAM: 6-carboxyfluorescein
FBS: fetal bovine serum
GAPDH: glyceraldehyde-3-phosphate dehydrogenase
GBM: glioblastoma multiforme
GTPase: guanosine triphosphatase
Heparin IU: Heparin International Unity
IVIS: In Vivo Imaging System
HEPES: *N*-2-hydroxyethylpiperazine-*N*′-2-ethanesulfonic acid
HCl: acid chloride
i.v: intravenously
KH: Krebs–Henseleit
LDH: lactate dehydrogenase
LINCS: Library of Integrated Network-Based Cellular Signatures
mA: milliampere
MGMT: O^6^-methylguanine-DNA methyltransferase
MD: molecular dynamics
MTORC1: mammalian target of rapamycin complex 1
NHS ester: *N*-hydroxysuccinimide ester
NMR: nuclear magnetic resonance
N/P: dendrimer protonable nitrogens/siRNA phosphorus
NTA: Nanoparticle Tracking Analyzer
p42-MAPK: p42 Mitogen-Activated Protein Kinase
PAMAM: poly­(amidoamine)
PBS: phosphate buffered saline
Rheb: RAS homologue enriched in brain
RNases: ribonucleases
RNA: ribonucleic acid
RNAi: interference ribonucleic acid
SASA: solvent-accessible surface area
SCR: scramble noncoding siRNA
SE: secondary electron detector
SEM: scanning electron microscopy
SEM: standard error of the mean
SDS-PAGE: sodium dodecyl sulfate polyacrylamide gel electrophoresis
STEM: scanning transmission wlectron detector
siRNA: small interfering ribonucleic acid
TAE: tris base, glacial acetic acid, ethylenediamine tetra-acetic acid
TEC: thiol–ene click chemistry
TMP: trimethylolpropane
TMZ: temozolamide
TIP3P water: 3-site rigid water
UV–vis: ultraviolet–visible spectroscopy
v/v: volume/volume
