# Supplementary material for: Hydrolytically Stable Cationic Bis-MPA Dendrimers as Efficient Transfectants for Glioblastoma Cells and Primary Astrocytes
Source: Biomacromolecules. 2025 Dec 3;27(1):234–48. doi: 10.1021/acs.biomac.5c01202 (PMC12801319; doi:10.1021/acs.biomac.5c01202)
Supplement: Supplementary file 1 [file bm5c01202_si_001.pdf]

Hydrolytically Stable Cationic bis-MPA Dendrimers as Efficient Transfectants for Glioblastoma Cells and Primary Astrocytes

Angel Buendía[1,2,†] , Natalia Sanz del Olmo[3†], Irene Rodríguez-Clemente[1,2], Jacob Wohlert[3,4], Krzysztof Sztandera[1,2], Jorge San Jacinto García[3], Faridah Namata[3], Michael Malkoch[3], Valentín Ceña[1,2*#]*

[1] Unidad Asociada Neurodeath. Institute of Molecular Nanoscience, INAMOL. School of Medicine; Universidad de Castilla-La Mancha, 02006 Albacete, Spain.

[2] CIBERNED, CIBER, Instituto de Salud Carlos III, Madrid, Spain.

[3] Department of Fiber and Polymer Technology, KTH Royal Institute of Technology, Teknikringen 56-68, 100 44, Stockholm, Sweden.

[4] Wallenberg Wood Science Centre (WWSC), KTH Royal Institute of Technology, 100 44 Stockholm, Sweden.

[†]Both authors contributed equally to this work

*Corresponding authors

[#]Address correspondence to:

Prof. Valentín Ceña
Universidad de Castilla-La Mancha
Unidad Asociada Neurodeath
Facultad de Medicina
Avda. Almansa, 14

02006 Albacete (SPAIN)
Email: valentin.cena@gmail.com
Telephone +34680222322

SUPPLEMENTARY INFORMATION

Index

1. Synthesis of TMP-G3-(Cys)23(Cy7.5)………………………………………… 3
2. Lack of transfection of G2-DA and G3-DA ………………………………….. 4
3. Lack of transfection of G1-CYS and G2-CYS ………………………………. 5
4. Toxicity of other transfectant agents………………………………………….. 6
5. Knockdown of p42-MAPK protein in mouse primary astrocytes……………. 7
6. Knockdown of p42-MAPK protein in human glioblastoma T98G cells……… 8

Synthesis of TMP-G3-(Cys)$_{23}$(Cy7.5).

In a round bottom flask equipped with a magnetic stirrer and protected from light, the dendrimer TMP-G3-(Cys)$_{24}$ (50.0 mg, 7.9 x 10^{-3} mmol) was dissolved in 1 mL DI water and the dye NHS activated Cy7.5 (6.6 mg, 7.9 x 10^{-3} mmol) was dissolved in 300 µL of DMSO and added to the reaction mixture. The reaction was kept stirring and protected from light at 50 ºC overnight. Afterwards, the mixture was dialyzed in DI water using a dialysis membrane of 1 kDa. The compound **TMP-G3-(Cys)$_{23}$(Cy7.5)** was obtained as a green solid (50.3 mg, 91 %). The completion of the reaction and purity of the reaction was confirmed through a ^{1}H-2D-DOSY NMR. ^{1}H-NMR (400 MHz, CD_3OD) δ/ppm: 8.33-6.20 (Cy7.5 signals), 4.42 – 4.16 (42H, m, H4, H8 and H12), 3.56 (96H, m, H16 and H17), 3.19 (46H, m, H21), 2.88 (46H, m, H20), 2.69 (48H, m, H19), 1.89 (48H, m, H18), 1.65 (2H, m, H2), 1.39 (9H, s, H7), 1.29 (18H, s, H11), 1.21 (36H, s, H15), 1.04 (3H, m, H1). UV-VIS (DI Water, 1 x 10 $^{-5}$ M), absorption maximum (nm): 800.

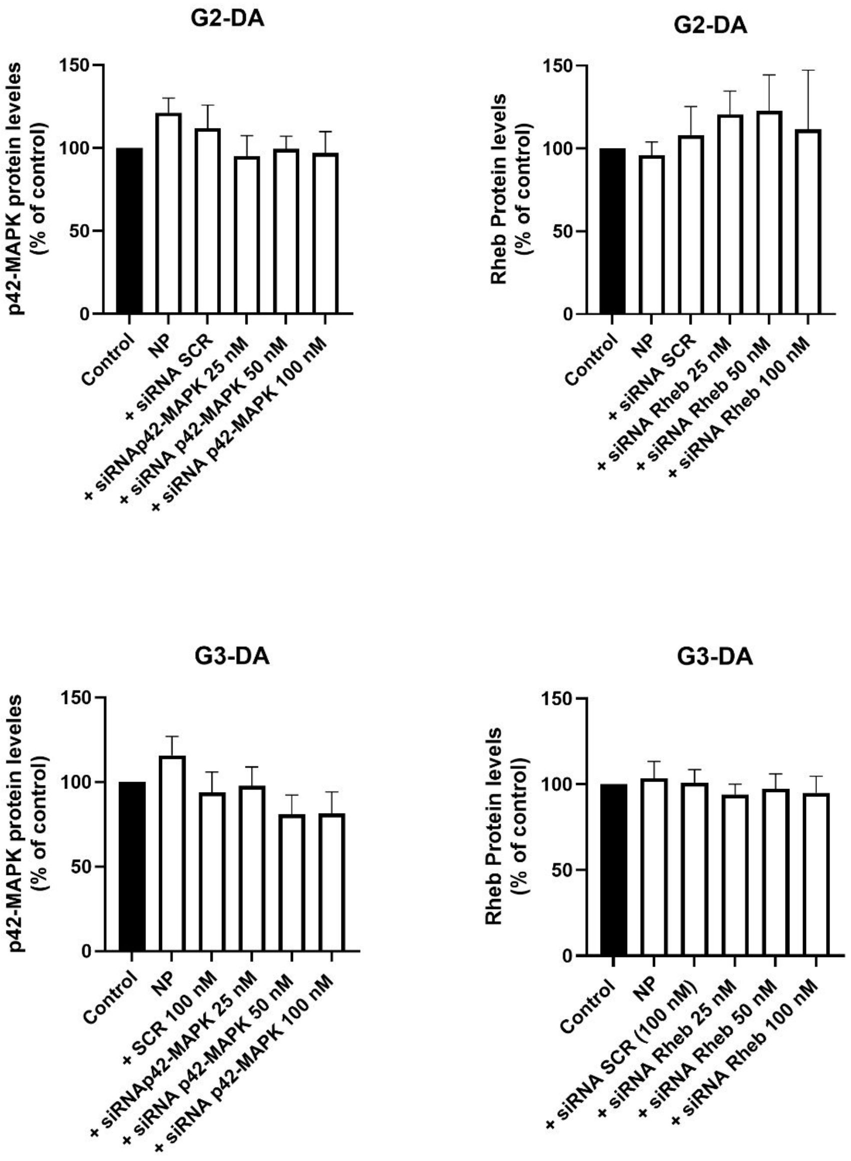

Figure SI1. Lack of transfection of p42-MAPK and Rheb, proteins in T98G GBM cells. Dendriplexes formed by 1 μM G2-DMA or G3-DMA and different concentrations of siRNA (25 to 100 nM) targeting either p42-MAPK or Rheb or Scramble (SCR) siRNA were incubated with T98G cells for 72 h. Then, cellular content was quantified as indicated in Experimental Procedure section. Data represent mean + S.E.M. of 4 to 6 experiments.

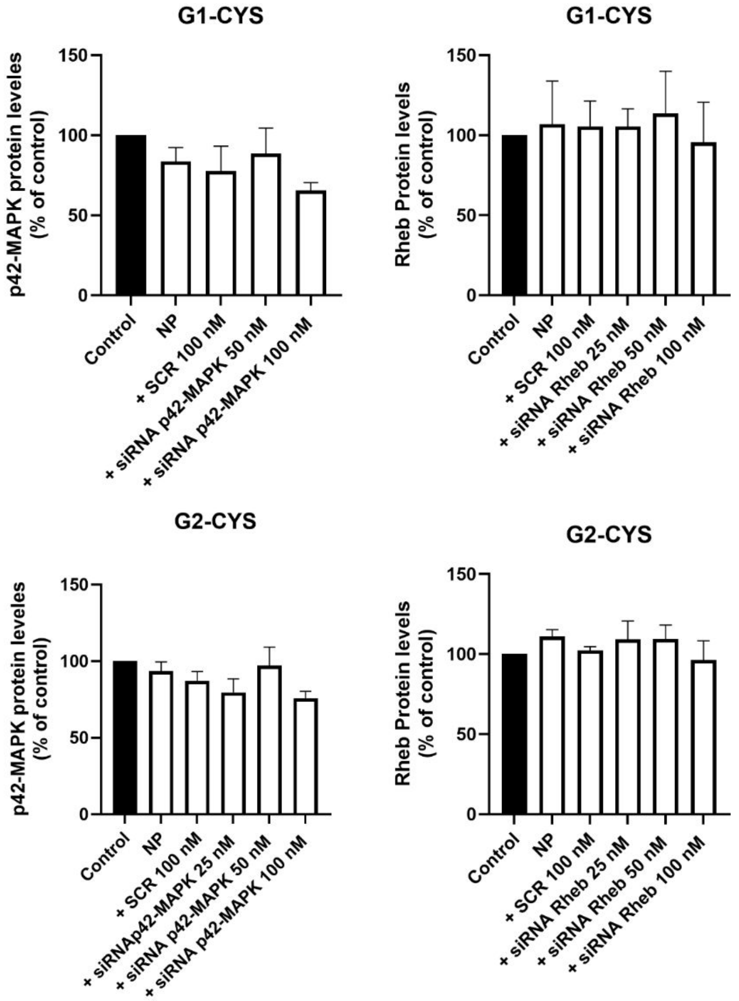

Figure SI2. Lack of transfection of p42-MAPK and Rheb, proteins in T98G GBM cells.

Dendriplexes formed by G1-CYS (5 μM) or G2-CYS (1 μM) and different concentrations of siRNA (25 to 100 nM) targeting either p42-MAPK or Rheb. Scramble (SCR) siRNA sequence was used as a control. Dendriplexes were incubated with T98G cells for 72 h. Then, cellular content was quantified as indicated in Experimental Procedure section. Data represent mean + + S.E.M. of 4 to 6 experiments.

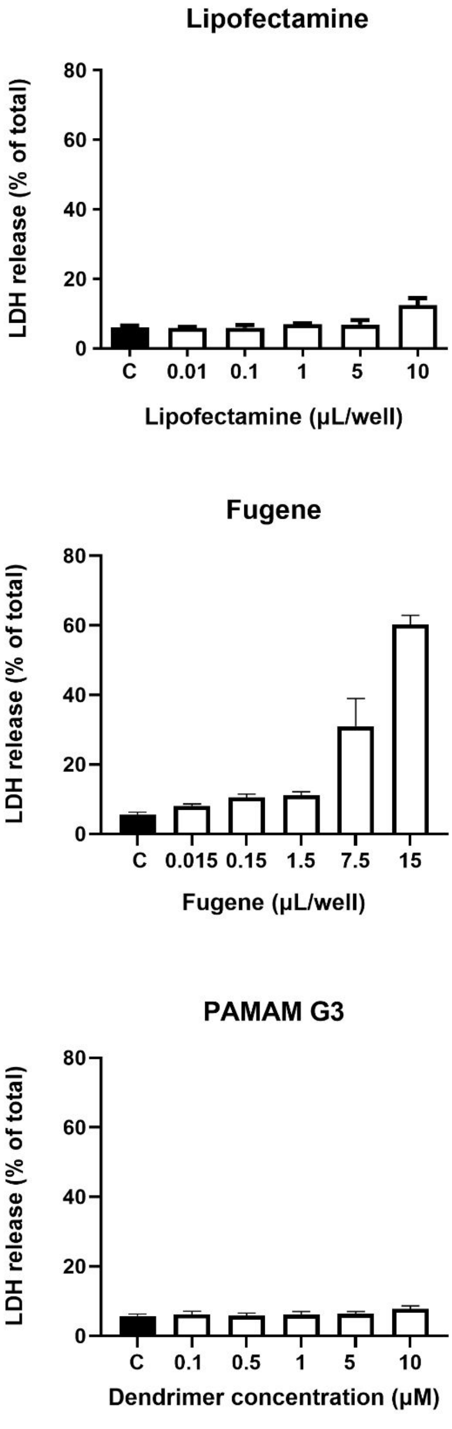

Figure SI3. **Toxicity of other transfectant agents.** T98G cells were exposed to the indicated concentrations of Lipofectamine RNAiMAX®, Fugene® or PAMAM G3 and toxicity determined as LDH release to the culture medium determined at 72 h. Data are reported as mean values, with error bars indicating the S.E.M for n=8 experiments.

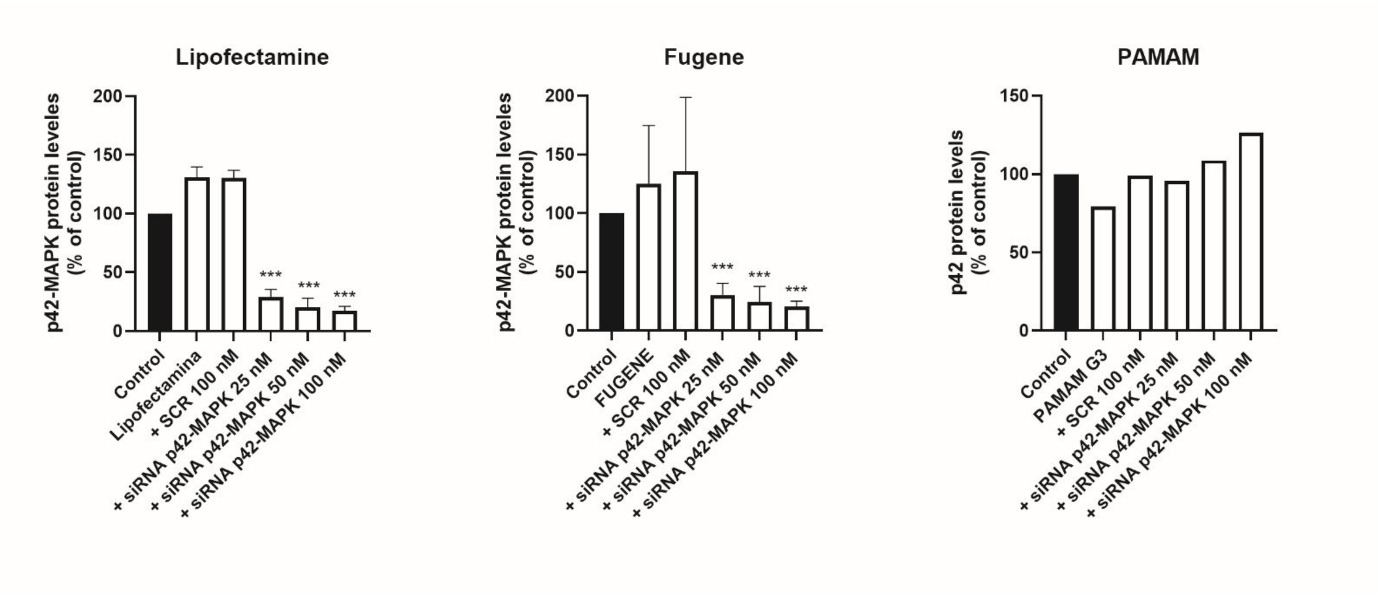

Figure SI4. Knockdown of p42-MAPK protein in mouse primary astrocytes. Complexes were formed by incubating; Lipofectamine RNAiMAX® (final concentration:1 µL/well; Fugene®; final concentration:1.5 µL/well), and PAMAM G3 (1 µM) with different concentrations of siRNA (25, 50 or 100 nM) targeting either p42-MAPK with primary astrocytes for 72 h. Cellular protein content was then quantified as described in the Experimental Procedures section. Data represent the mean with error bars indicating the S.E.M. of 2 experiments. ***$p < 0.001$ when compared to control.

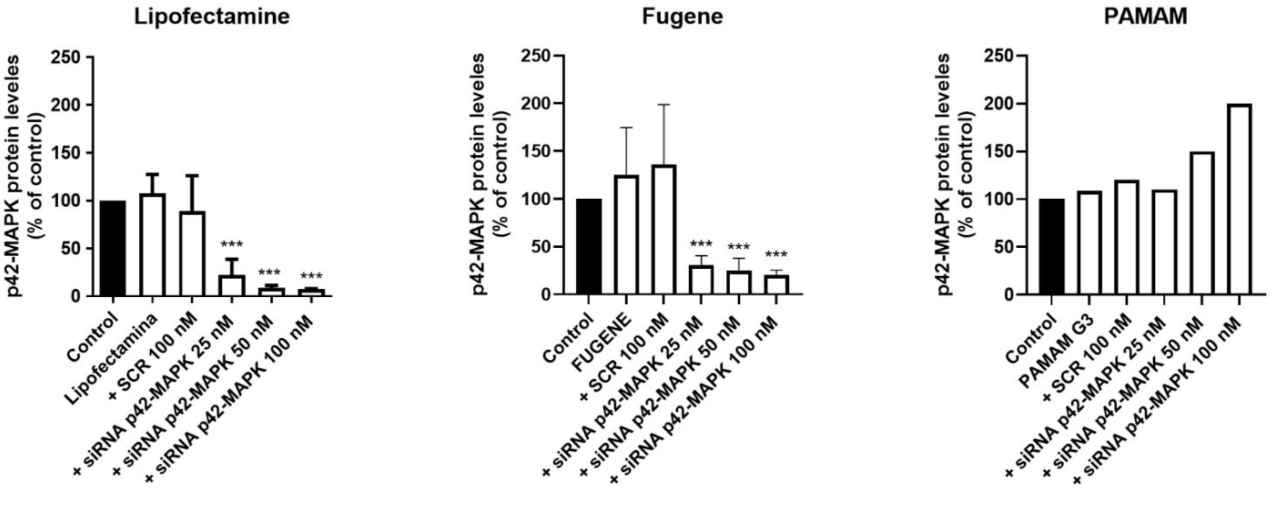

Figure SI5. Knockdown of p42-MAPK protein in human glioblastoma T98G cells.

Complexes were formed by incubating; Lipofectamine RNAiMAX® (final concentration:1 µL/well); Fugene®; final concentration:1.5 µL/well), and PAMAM G3 (1 µM) with different concentrations of siRNA (25, 50 or 100 nM) targeting either p42-MAPK with T98G cells for 72 h. Cellular protein content was then quantified as described in the Experimental Procedures section. Data represent the mean with error bars indicating the S.E.M. of 2 experiments. ***$p < 0.001$ when compared to control.
